# Intrinsic brain functional connectivity predicts treatment-related motor complications in early Parkinson’s disease patients

**DOI:** 10.1007/s00415-023-12020-6

**Published:** 2023-10-09

**Authors:** Rosa De Micco, Federica Di Nardo, Mattia Siciliano, Marcello Silvestro, Antonio Russo, Mario Cirillo, Gioacchino Tedeschi, Fabrizio Esposito, Alessandro Tessitore

**Affiliations:** 1 Department of Advanced Medical and Surgical Sciences, University of Campania “Luigi Vanvitelli”, Naples, Italy; 2 Neuropsychology Laboratory, Department of Psychology, University of Campania “Luigi Vanvitelli”, Caserta, Italy

**Keywords:** Parkinson’s disease, Drug-naïve, Treatment-related complications, Resting-state connectivity, MRI

## Abstract

**Background:**

Treatment-related motor complications may develop progressively over the course of Parkinson’s disease (PD).

**Objective:**

We investigated intrinsic brain networks functional connectivity (FC) at baseline in a cohort of early PD patients which successively developed treatment-related motor complications over 4 years.

**Methods:**

Baseline MRI images of 88 drug-naïve PD patients and 20 healthy controls were analyzed. After the baseline assessments, all PD patients were prescribed with dopaminergic treatment and yearly clinically re-assessed. At the 4-year follow-up, 36 patients have developed treatment-related motor complications (PD-Compl) whereas 52 had not (PD-no-Compl). Single-subject and group-level independent component analyses were used to investigate FC changes within the major large-scale resting-state networks at baseline. A multivariate Cox regression model was used to explore baseline predictors of treatment-related motor complications at 4-year follow-up.

**Results:**

At baseline, an increased FC in the right middle frontal gyrus within the frontoparietal network as well as a decreased connectivity in the left cuneus within the default-mode network were detected in PD-Compl compared with PD-no-Compl. PD-Compl patients showed a preserved sensorimotor FC compared to controls. FC differences were found to be independent predictors of treatment-related motor complications over time.

**Conclusion:**

Our findings demonstrated that specific FC differences may characterize drug-naïve PD patients more prone to develop treatment-related complications. These findings may reflect the presence of an intrinsic vulnerability across frontal and prefrontal circuits, which may be potentially targeted as a future biomarker in clinical trials.

**Supplementary Information:**

The online version contains supplementary material available at 10.1007/s00415-023-12020-6.

## Introduction

Despite considerable advances in pharmacotherapy, levodopa remains the mainstay for treatment of Parkinson disease (PD) due to its superior efficacy [1]. However, long-term levodopa stimulation is associated with the development of treatment-related motor complications, mainly motor fluctuations and levodopa-induced dyskinesia (LID). These disabling phenomena occur in 40–60% of patients with PD after 5 years of treatment [2] and can markedly impair patients’ quality of life [2].

Several factors have been associated with later treatment-related motor complications, yet not able to comprehensively explain their risk over time [1–3]. A combined effect of chronic dopaminergic stimulation together with intrinsic biological factors such as sex, age, genetic expression profile, and the progressive neuronal alterations induced by the neurodegeneration has been proposed to be involved in the development of these troublesome symptoms [2].

Individual prediction of motor complications would be dramatically helpful for therapeutic management in patients with PD, particularly since treatment options are limited.

Functional MRI (fMRI) provides evidence of brain neural connectivity and plasticity by means of the blood oxygenation level-dependent phenomenon [4]. When fMRI sequences are acquired at rest (i.e., resting-state fMRI, rs-fMRI), it is possible to map the spontaneous fluctuations of the BOLD signals which synchronically coactivated in different brain regions over specific time series within the so-called resting-state functional networks (RSNs) [4]. Evidence that the reorganization of RSNs occurs in aging and in pathological brains [4, 5] even before neuronal death or brain atrophy suggests a potential for rs-fMRI to provide sensitive and early markers of neurodegenerative processes.

Previous MRI studies in patients with treatment-related motor complications showed the presence of disrupted functional and structural connectivity within the cortico-striatal pathway [6]. However, the modulatory effect of dopaminergic treatment over this network has been consistently shown in PD patients, with and without motor complications [6]. Thus, while these studies may provide evidence of disease- and treatment-related connectivity changes occurring in the brain at the time of complications development, it is yet not possible to infer whether those differences represent an intrinsic predisposing vulnerability to be potentially anticipated.

Two PET studies showed that higher dopaminergic levels and turnover may predict the future development of motor complications in early PD patients [7, 8]. In previous studies, we have demonstrated the presence of early connectivity differences within frontal and frontostriatal circuits in drug-naïve PD patients [9–11], also showing a specific divergence by sex [10] and sensitivity to dopaminergic treatments [9].

In this study, we aimed to investigate whether baseline functional connectivity patterns over motor and non-motor networks may be associated to later treatment-related motor complications in a longitudinal cohort of drug-naïve PD patients. We hypothesize that early dysfunctional or even compensatory deviations may be detected in the early stages of PD and may represent a disease-intrinsic predisposing factor likely interplaying with dopaminergic treatment and other neurodegeneration-related processes occurring over the disease course. This unique architecture may potentially be used to predict disease progression in the early stages of the disease and to foster individualized treatment strategies.

## Materials and methods

### Study population

The study sample was recruited from an ongoing longitudinal project enrolling consecutive patients with early PD diagnosed according to the modified diagnostic criteria of the UK Parkinson’s Disease Society Brain Bank [12] at the Movement Disorders Unit of the First Division of Neurology at the University of Campania “Luigi Vanvitelli” (Naples, Italy). As previously described [10, 11], patients enrolled in this longitudinal cohort underwent an extensive motor and non-motor assessment at the time they were diagnosed with PD. After the baseline assessments, patients were prospectively followed with a full clinical evaluation every year.

In this study, drug-naïve PD patients with a modified Hoehn and Yahr (mH&Y) [13] stage ≤ 2.5 at baseline were included. Exclusion criteria were: (1) PD onset before age 40 years; (2) any previous treatment with dopaminergic, anticholinergic, antidepressant, or other centrally acting drugs, to rule out a potential effect on functional connectivity from these agents; (3) relevant cognitive impairment associated with PD according to consensus criteria [14, 15]; and (4) any other neurological disorder or clinically significant medical condition. Moreover, 20 healthy age- and sex-matched healthy controls (HC), with no familiar history of PD nor parkinsonism, were also enrolled.

All the subjects signed their written informed consent prior the inclusion in the study. The study was approved by the ethics committee of the University of Campania “Luigi Vanvitelli”, Naples, Italy.

### Study design

At baseline, we collected clinical and imaging data in the morning, in the same day, in distinct sessions. One week after the baseline assessments, all patients started a dopaminergic treatment which was determined according to their motor status by two trained clinicians, following current international guidelines [16]. Then PD patients were followed for an observation period, lasting 4 years, undergoing an extensive clinical follow-up every 12 months.

At each follow-up visit, motor features were re-assessed by the same clinicians, and treatment details were registered. According to the motor outcome of each patient and to the presence of complications, dopaminergic regimen was modified as needed.

Each patient who was diagnosed with treatment-related motor complications (as described below), at the time of any follow-up visit, was sub-grouped as PD-Compl and discharged from the observation period. Patients with PD who did not develop any treatment-related motor complications throughout the observation period of 4 years were sub-grouped as PD-no-Compl.

### Clinical motor and neuropsychological assessments

Disease severity and motor performance were assessed using the mH&Y stages [13] and the Unified Parkinson’s Disease Rating Scale part III (UPDRS III) [17], at baseline and at each follow-up visit. Moreover, the presence of any treatment-related motor complications was assessed by means of the UPDRS part IV [17] (A-B subscales). Patients were considered to present motor complications if the total score of A-B subscales was ≥ 1. At each follow-up visit, the levodopa equivalent daily dose (LEDD) was calculated for both dopamine agonists (LEDD-DA) and dopamine agonists + L-dopa (total LEDD) [18].

Global cognition in patients and HC was assessed by means of Montreal Cognitive Assessment (MoCA) [14, 19] and Mini-Mental State Examination (MMSE) [20], respectively. All patients performed a comprehensive neuropsychological battery including tests to explore the following cognitive domains: *attention and working memory* (digit span backward [21, 22], and Trail Making Test-A [23, 24]), *memory* (Rey’s Auditory Verbal Learning Test—delayed recall [25, 26], and prose recall test [27, 28]), *executive functions* (letter fluency task [26, 29] and Modified Card Sorting Test [30, 31], number of achieved categories), *visuospatial abilities* (Judgment of Line Orientation test [32, 33] and drawing copying test [34]), and *language* (noun and verb denomination task [35]). Moreover, for noun and verb denomination tasks, we used internal normative data developed on 59 healthy controls (age: 61.50 ± 9.93; education: 12.89 ± 3.77; male/female: 22/37) who were free from cognitive impairment (all had age- and education-adjusted MoCA scores higher than Italian cutoff score (15.5) [19]), and were matched to patients for age and education. Finally, cognitive domain z-scores (i.e., *z*-score executive, z-score attention/working memory, *z*-score visuospatial, *z*-score memory, *z*-score language) were computed by averaging *z*-scores of the neuropsychological tests included in the same domain.

### Imaging parameters

Magnetic resonance images were acquired on a General Electric 3 Tesla MRI scanner equipped with an eight-channel parallel head coil. A 6-min fMRI sequence was acquired, consisting of 240 volumes of a repeated gradient-echo echo planar imaging T2*-weighted sequence (TR = 1508 ms, axial slices = 29, matri*x = *64 × 64, field of view = 256 mm, thickness = 4 mm, interslice ga*p = *0 mm). During the functional scan, subjects were asked to simply stay motionless, awake, with their eyes closed. Three-dimensional high-resolution T1-weighted sagittal images (GE sequence IR-FSPGR, TR = 6988 ms, TI = 1100 ms, TE = 3.9 ms, flip angle = 10, voxel size = 1 × 1 × 1.2 mm^3^) were acquired for registration and normalization of the functional images as well as for voxel-based morphometry (VBM) analysis.

### Resting-state fMRI pre-processing and statistical analysis

Image data pre-processing and statistical analysis were performed with BrainVoyager QX (Brain Innovation BV, The Netherlands). Before statistical analyses, translational motion parameters were verified to be always less than 1 functional voxel for all included participants. We further verified that there were no statistically significant differences in the mean frame-wise displacement (a surrogate metric of head motion accounting for intra-voxel residual motion effects) when carrying group comparisons and that adjusting the scores for mean frame-wise displacement did not affect the significance of the group effects. Then individual functional data were co-registered to their own anatomical data and spatially normalized to the standard Talairach space. Single-subject independent component analysis (ICA) was carried out on each pre-processed fMRI time series with the fastICA [36]. The ICASSO step was added to the extraction of single-subject ICA components: ICASSO [37] is a validated procedure to ensure the maximal algorithmic and statistical stability of ICA components of neuroimaging time series and entails with running the FastICA algorithm many times (in our study, we set the number of repetitions to 20) with different initial values (algorithmic reliability) and with differently bootstrapped data sets (statistical reliability).

For each subject, 40 ICA components were extracted. The best matching between each single-subject ICA component map and the network masks of the main physiological RSNs (as derived from an external HC group) was determined from the highest goodness-of-fit, i.e., the highest difference between the average ICA score inside the mask and the average ICA score outside the mask [38]. Here we considered the most reported and investigated RSNs, i.e., the default-mode network (DMN), the sensorimotor network (SMN), the frontoparietal network (FPN), the ventral (VAN), and dorsal-attention (DAN) networks [39].

For homologue components corresponding to a given RSN template mask, a multi-subject random effects (RFX) analysis was carried out that treated the individual subject map values as random observations at each voxel. The network masks were also applied to define the search volume for within-network group comparisons in a voxel-wise analysis of the ICA scores (i.e., PD-Compl vs. PD-no-Compl; PD-Compl vs. HC; PD-no-Compl vs. HC). Then, a two-sample *t* test was computed at each voxel of the mask to produce a t-map of the differences. To correct for multiple comparisons across all voxel-wise comparisons, regional effects were only accepted for clusters of voxels exceeding a minimum size determined with a non-parametric randomization approach. Namely, an initial voxel-level threshold was set to *p < *0.05 (uncorrected) and then a minimum cluster size was estimated after 1000 Monte Carlo simulations that protected against false positive clusters up to 5%. Sex effects were considered as covariates of no interest and linearly regressed out from the series of maps prior to statistical comparisons.

### VBM analysis

Data were processed and examined using SPM12 software (Wellcome Trust Centre for Neuroimaging, London, UK; http://www.fil.ion.ucl.ac.uk/spm), with default parameters incorporating the DARTEL toolbox, which was used to obtain a high-dimensional normalization protocol [40]. Images were bias-corrected, tissue-classified, and registered using linear (12-parameter affine) and non-linear transformations (warping) within a unified model. Subsequently, the warped grey matter (GM) segments were affine-transformed into Montreal Neurological Institute (MNI) space and were scaled by the Jacobian determinants of the deformations to account for the local compression and stretching that occurs as a consequence of the warping and affine transformation (modulated GM volumes). Finally, the modulated volumes were smoothed with a Gaussian kernel of 8-mm full-width at half maximum (FWHM). The GM volume maps were statistically analyzed using the general linear model based on Gaussian random field theory. Statistical analysis consisted of an ANCOVA with total intracranial volume (TIV) and age as covariates of no interest. Statistical inference was performed at the voxel level, with a family-wise error (FWE) correction for multiple comparisons (*p < *0.05).

### Statistical analysis of clinical, motor, and neuropsychological data

Demographic data between PD sub-groups and controls were compared using ANOVA models. *t* test was used to compare clinical variables between PD-Compl and PD-no-Compl sub-groups. Chi-square was used to determine differences in the distribution of categorical variables. Analyses were all Bonferroni corrected for multiple comparisons. To determine the independent predictors of treatment-related motor complications over time, univariate Cox regression was run including clinical (i.e., age, sex, disease duration and UPDRSIII at baseline and total LEDD at the end of the observation period) and functional imaging measures (i.e., average ICA scores). Factors significantly associated with the development of treatment-related motor complications at the 4-year follow-up in the univariate model as well as on the basis of clinical interest were included in a multivariate regression model. A *p < *0.05 was considered statistically significant. Analyses were performed with SPSS version 20 (SPSS Inc. Chicago, IL).

## Results

### Clinical findings

One hundred fifty-one drug-naïve PD patients were initially enrolled in the study. Four patients were excluded as they received an alternative diagnosis over the clinical observation (two subjects were diagnosed with multiple system atrophy; two cases with dementia with Lewy body). Over the observation period, 36 PD patients developed treatment-related motor complications and were labeled as PD-Compl. In detail, 9 PD-Compl patients had only LID, while 17 had only motor fluctuations. Ten patients presented both LID and other motor fluctuations. Fifty-two PD patients did not develop motor complications and agreed to complete the 4-year assessments, and were, therefore, labeled as PD-no-Compl. We also enrolled 20 age- and sex-matched HC. At baseline, no significant demographic and clinical differences were detected between the two patients sub-groups (i.e., PD-Compl and PD-no-Compl). No differences in terms of LEDD and LEDD-DA were detected between PD-Compl and PD-no-Compl at the end of the observation period. Clinical and demographic characteristics of patient sub-groups at baseline and at follow-up are shown in Table 1.

**Table 1:** Demographic and clinical features of PD patients and controls at baseline and at the end of the observation period

Variable	HC (*n = *20) mean ± SD	PD-Compl (*n = *36) mean ± SD	PD-no-Compl (*n = *52) mean ± SD	*p* value
Baseline				
Age (y)	58.90 ± 5.83	59.92 ± 8.40	63.13 ± 9.04	0.96
Education (y)	11.31 ± 3.90	10.36 ± 4.21	10.52 ± 3.79	1.00
Sex (M/F)	12/8	14/22	31/21	1.00
Disease duration (m)	–	17.67 ± 6.52	14.65 ± 8.04	0.72
mH&Y	–	1.43 ± 0.54	1.48 ± 0.53	1.00
UPDRS III	–	19.72 ± 6.78	17.75 ± 8.07	1.00
MoCA	–	23.47 ± 4.28	23.20 ± 3.45	1.00
MMSE	28.5 ± 1.12	–	–	–
*z*-score attention/WM	–	− 0.30 ± 0.77	− 0.43 ± 0.98	1.00
*z*-score memory	–	− 0.92 ± 0.73	− 0.90 ± 0.78	1.00
*z*-score executive	–	− 0.29 ± 0.62	− 0.70 ± 1.20	1.00
*z*-score language	–	− 0.02 ± 0.67	− 0.04 ± 0.49	1.00
*z*-score visuospatial	–	− 1.15 ± 0.70	− 1.20 ± 1.32	1.00
Follow-up				
Dyskinesia (yes/no)	–	19/17	–	–
Wearing-off (yes/no)	–	27/9	–	–
UPDRS IV	–	4.55 ± 1.61	–	–
Time to motor complications (m)	–	33.78 ± 15.80	–	–
L-Dopa at treatment initiation (yes/no)	–	13/23	28/24	0.30
LEDD-dopa (end of study)	–	326.39 ± 107.39	273.08 ± 170.19	0.30
LEDD-DA (end of study)	–	68.19 ± 116.43	69.77 ± 97.46	1.00

*p* values refer to ANOVA models, pairwise *t* test or *χ*^2^ as appropriate. Analyses were also Bonferroni corrected for multiple comparisons (for baseline variables 0.004 (0.05/12); for follow-up variables 0.02 (0.05/3)

*PD* Parkinson’s disease; *HC* healthy controls; *SD*  standard deviation; *m* months; *mH&Y  *modified Hoehn and Yahr; *UPDRS* Unified Parkinson’s Disease Rating Scale; *MoCA* Montreal Cognitive Assessment; *MMSE* Mini-Mental State Examination; *WM* working memory; *LEDD* levodopa equivalent dose; *DA* dopamine agonist

.

### Imaging findings

#### Functional connectivity

##### PD-Compl vs. PD-no-Compl (Fig. 1)

**Fig. 1:**
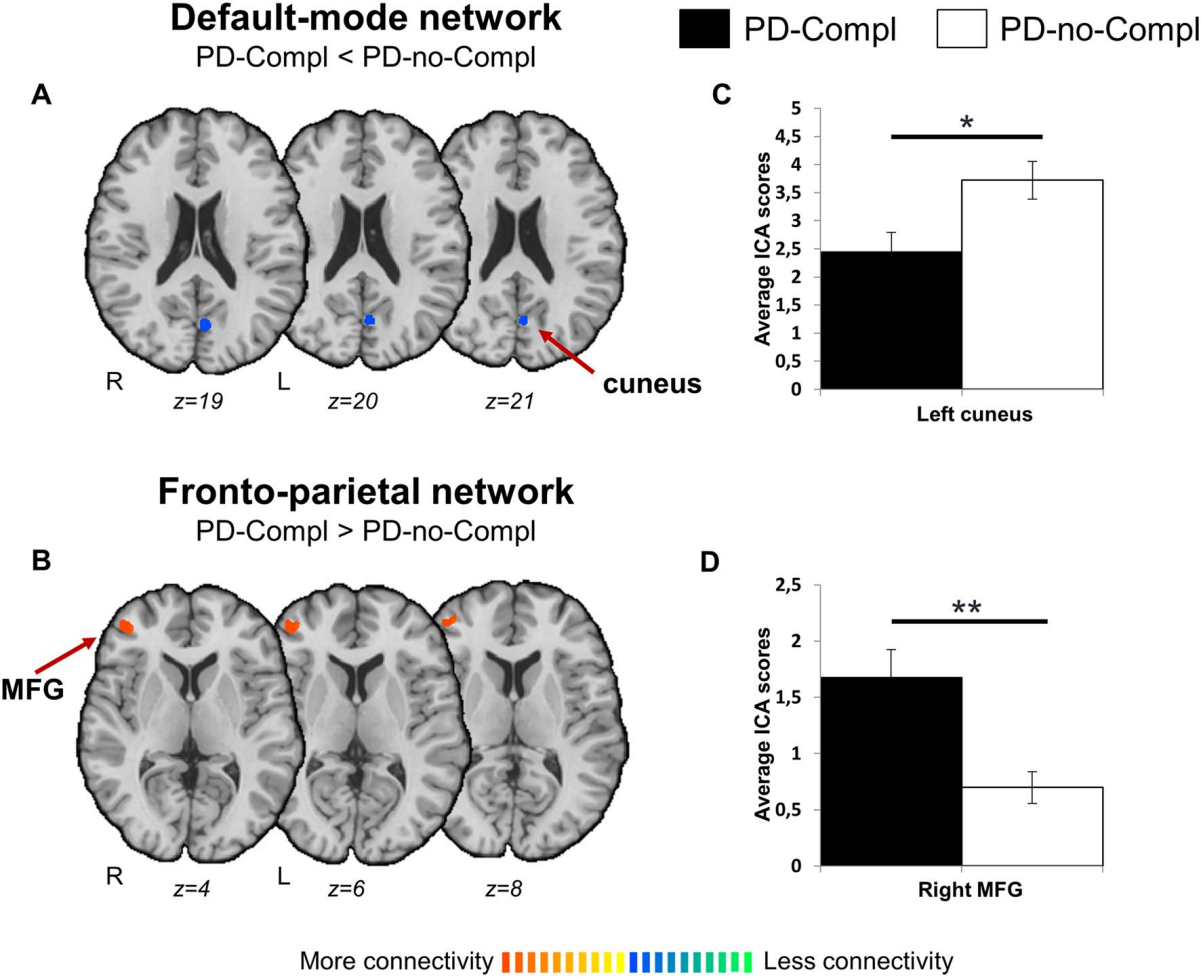
Resting-state network connectivity differences in PD patients with and without treatment-related motor complications. **A**, **B** Whole-network significant connectivity differences between PD-Compl and PD-no-Compl patients. **C**, **D** Bar plots of the average ICA scores within the default-mode and the frontoparietal networks (**p < 0.001; *p < 0.05). Cold colors represent less, and hot colors represent more connectivity. *ICA* independent component analysis; *R* right; *L* left, *MFG* middle frontal gyrus

Compared to PD-no-Compl, PD-Compl showed an increased functional connectivity in the right middle frontal gyrus (MFG, *x = *39; *y = *46; *z = *10) within the FPN as well as a decreased functional connectivity in the left cuneus (*x = *− 3; *y = *− 70; *z = *19) within the DMN (*p < *0.05, corrected for multiple comparisons).

##### PD-Compl vs. HC (Supplementary Fig. 1)

Compared to HC, PD-Compl showed a decreased connectivity in the left superior parietal lobule within the DAN (*x = *− 16; *y = *− 67; *z = *55); a decreased connectivity in the left medial frontal gyrus within the DMN (*x = *− 7; *y = *62; *z = *1); an increased functional connectivity in the right MFG (*x = *39; *y = *49; *z = *13) within the FPN; a decreased connectivity in the right medial frontal gyrus within the VAN (*x = *12; *y = *5; *z = *55). No differences were detected between PD-Compl patients and HC within the SMN (*p < *0.05, corrected for multiple comparisons).

##### PD-no-Compl vs. HC (Supplementary Fig. 2)

Compared to HC, PD-no-Compl showed an increased connectivity within the right precuneus (*x = *15; *y = *− 67; *z = *46) as well as a decreased connectivity in the left superior parietal lobule (*x = *− 27; *y = *− 67; *z = *58) within the DAN; a decreased connectivity in the left medial frontal gyrus (*x = *− 6; *y = *62; *z = *4) within the DMN; a decreased connectivity in the left angular gyrus within the FPN (*x = *− 46; *y = *− 70; *z = *37); a decreased connectivity in the left paracentral lobule within the SMN (*x = *− 3; *y = *− 11; *z = *46); a decreased connectivity in anterior cingulate cortex within the VAN (*x = *− 6; *y = *5; *z = *46) (*p < *0.05, corrected for multiple comparisons).

#### VBM

No statistically significant differences in local gray matter atrophy were found between the two PD sub-groups and between all patients and HC (*p < *0.05 FWE).

### Regression analyses

Univariate and multivariate (including age, sex, disease duration, and UPDRSIII at baseline, total LEDD at the end of the observation period) Cox regression models indicated that functional connectivity within the MFG (*x = *39; *y = *46; *z = *10) and the left cuneus (*x = *− 3; *y = *− 70; *z = *19) were independent predictors of treatment-related motor complications at 4-year follow-up in PD patients (*p ≤ *0.001 and *p = *0.005, respectively) (Table 2).

**Table 2:** Baseline predictors of treatment-related motor complications at 4-year follow-up in PD patients

	PD patients (*n = *88)			
	*Univariate analysis*	*Multivariate* *analysis*	*Coefficient*	*Hazard ratio*
	*p* value	*p* value		
**Demographic and clinical variables**				
Age	0.897	0.349	0.025	1.025
Sex	0.109	0.213	0.504	1.656
Disease duration at baseline	0.459	0.565	− 0.016	0.985
UPDRS III at baseline	0.195	0.232	0.037	1.037
Total LEDD (end of study)	0.641	0.561	− 0.001	0.999
**Functional connectivity PD-Compl vs. PD-no-Compl (average ICA scores)**				
FPN—right middle frontal gyrus	** < 0.001**	** < 0.001**	0.615	1.849
DMN—left cuneus	**0.033**	**0.005**	− 0.278	0.757

Significant differences are reported in bold

*PD-Compl* Parkinson’s disease patients with treatment-related motor complications; *LEDD* levodopa equivalent daily dose; *UPDRS* Unified Parkinson’s disease Rating Scale; *DMN *default-mode network; FP*N *frontoparietal network

## Discussion

In this study, we analyzed the brain functional architecture within motor and non-motor networks at the disease onset in a cohort of drug-naïve PD patients who eventually developed treatment-related complications. Even before starting any dopaminergic treatment, PD-Compl patients showed: (i) an increased functional connectivity within the FPN compared to PD-no-Compl patients and controls; (ii) a decreased functional connectivity within the DMN compared to PD-no-Compl patients; (iii) a preserved functional connectivity within the SMN compared to controls.

Moreover, functional connectivity within the FPN and the DMN was found to be an independent predictor of treatment-related motor complications over a 4-year follow-up period.

We found an increased functional connectivity in the MFG within the FPN in PD-Compl patients compared to PD-no-Compl and HC. The FPN has a pivotal role as a flexible hub for coordinating the activity of other brain networks and is critical for our ability to coordinate behavior in a rapid, accurate, and flexible goal-driven manner [41]. Even though with a degree of heterogeneity among studies, the FPN mainly maps in frontal and parietal association cortices [41]. Among other cortical areas, the MFG has been shown to be involved in motor task preparation [42]. In previous studies, LID have been linked to a neurochemical cascade triggered by drug-induced pulsatile stimulation of dopamine receptors but also involving downstream changes in genes expression, and abnormalities in non-dopaminergic transmitter systems [2–6]. All these phenomena eventually interfere with the firing pattern from the basal ganglia over the cortex, leading to excessive disinhibition of thalamocortical neurons and overactivation of frontal areas, including motor, premotor, and prefrontal cortices [43]. Thus, we hypothesize that an increased connectivity within the FPN, occurring early in the disease course, may be considered a disease-intrinsic predisposing factor over this frontal overdrive, eventually promoting the development of treatment-related motor complications under chronic dopaminergic stimulation. This parallels with a recent study showing the presence of white matter differences within frontostriatal and parietal regions in drug-naïve PD patients more vulnerable to develop LID over 5 years [44]. Our regression analysis further corroborate this hypothesis showing that functional connectivity within the MFG may predict the development of treatment-related motor complications at 4-year follow-up in PD patients even after accounting for clinical relevant factors such as age, sex, disease severity, and dopaminergic treatment.

Within the DMN, we revealed a decreased connectivity in the cuneus in PD-Compl compared to PD-no-Compl patients. The cuneus is crucial for visuospatial attention/working memory, but it is also implicated in visuomotor integration [45]. Interestingly, significant local grey matter atrophy of this area has been shown to differentiate schizophrenic patients with tardive dyskinesia from those without [45, 46]. An aberrant functional connectivity within the DMN has been found to be the most consistent finding to differentiate PD patients with and without cognitive impairment [47]. However, recent studies have shown that functional connectivity within the DMN may be modulated and restored by dopaminergic treatment [48]. An increased effect of dopaminergic medications on the DMN functional dynamics has been found in PD patients without LID compared to those with LID [49]. Based on these findings, we may hypothesize that the presence of a dysfunctional DMN connectivity could be present even before treatment initiation, as we found in our cohort in PD-Compl patients, but later on, it may be affected from chronic dopaminergic stimulation.

We found a decreased functional connectivity in the paracentral lobule within the SMN in PD-no-Compl compared to controls. This region is directly involved in the control of voluntary movements and sensory innervations [50] and it has been already found to be functionally disrupted in patients with PD [51]. Similarly, functional connectivity changes within the SMN have been consistently shown in PD patients all across disease stages [5]. This is quite reasonable as many SMN key nodes are direct targets of early PD-related neurodegenerative processes leading to the development of motor symptoms. Surprisingly, we did not find any SMN connectivity differences in PD-Compl patients compared to controls, likely suggesting that patients more prone to develop treatment-related motor complications may present at the time of diagnosis a more preserved SMN connectivity compared to others.

All in all, an abnormal connectivity within frontal and prefrontal areas could reflect the presence of synaptic remodeling and plasticity phenomena, perhaps following the presence of intrinsic biological factors, that may arise in the early stage of the disease and eventually lead to an increased cortico-striato-cortical firing, which have been related to the development of motor complications [2, 6, 52]. An abnormal dopaminergic modulation of resting‐state connectivity between the sensorimotor areas and the putamen has been indeed found to be associated with the development of LID in PD patients with treatment-related motor complications at the time of the fMRI data collection [53].

Our study has some limitations. We included, in the same PD sub-group (namely the PD-Compl), patients with both motor fluctuations (i.e., wearing-off phenomena) and LID. Study comparisons were not performed separately for patients presenting with LID (*n = *9) or motor fluctuations alone (*n = *17) as for their relatively small sample size. However, it is noteworthy that these phenomena are correlated and often detected together in the same patients [1], and this is reflected also in our cohort.

A longer follow-up would have allowed us to exclude the later development of treatment-related motor complications in other PD patients which have been labeled as PD-no-Compl after 4 years. On the other hand, by including only subjects presenting motor complications within the first 4 years of treatment, we had more chance to select more vulnerable patients irrespectively from the chronic long-term effect of dopaminergic medications.

Finally, a comparison with patients not taking dopaminergic medications and a longitudinal MRI assessments would have also provided more insights into the possible interplay between dopaminergic treatment, PD-related neurodegenerative processes, and intrinsic susceptibility to develop motor complications at a patient level.

These limitations notwithstanding our findings suggest the presence of early functional connectivity differences characterizing drug-naïve PD patients more prone to develop treatment-related complications over time.

This pattern may reflect the presence of a specific vulnerability across frontal and prefrontal circuits likely interplaying with dopaminergic treatment and other neurodegeneration-related processes occurring over the disease course and may be potentially targeted as a future biomarker in clinical trials. Future studies including larger PD populations are needed to support our observations.

### Supplementary Information

Below is the link to the electronic supplementary material.

Supplementary file1 Supplementary figure 1 Resting-state network connectivity changes in PD patients and controls. Whole-brain significant connectivity differences between PD-Compl and healthy controls. Cold colors represent less, and hot colors represent more connectivity. Abbreviations: R: right; L: left; MFG: middle frontal gyrus; medialFG: medial frontal gyrus; SPL: superior parietal lobule (TIF 2286 KB)

Supplementary file2 Supplementary figure 2 Resting-state network connectivity changes in PD patients and controls. Whole-brain significant connectivity differences between PD-no-Compl and healthy controls. Cold colors represent less, and hot colors represent more connectivity. Abbreviations: R: right; L: left; medialFG: medial frontal gyrus; ACC: anterior cingulate cortex; SPL: superior parietal lobule (TIF 2958 KB)

## Data Availability

The datasets used and/or analyzed during the current study are available from the corresponding author on reasonable request.
