# Supplementary figures and images for: Intrinsic brain functional connectivity predicts treatment-related motor complications in early Parkinson’s disease patients
Source: J Neurol. 2023 Oct 9;271(2):826–34. doi: 10.1007/s00415-023-12020-6 (PMC10827831; doi:10.1007/s00415-023-12020-6)

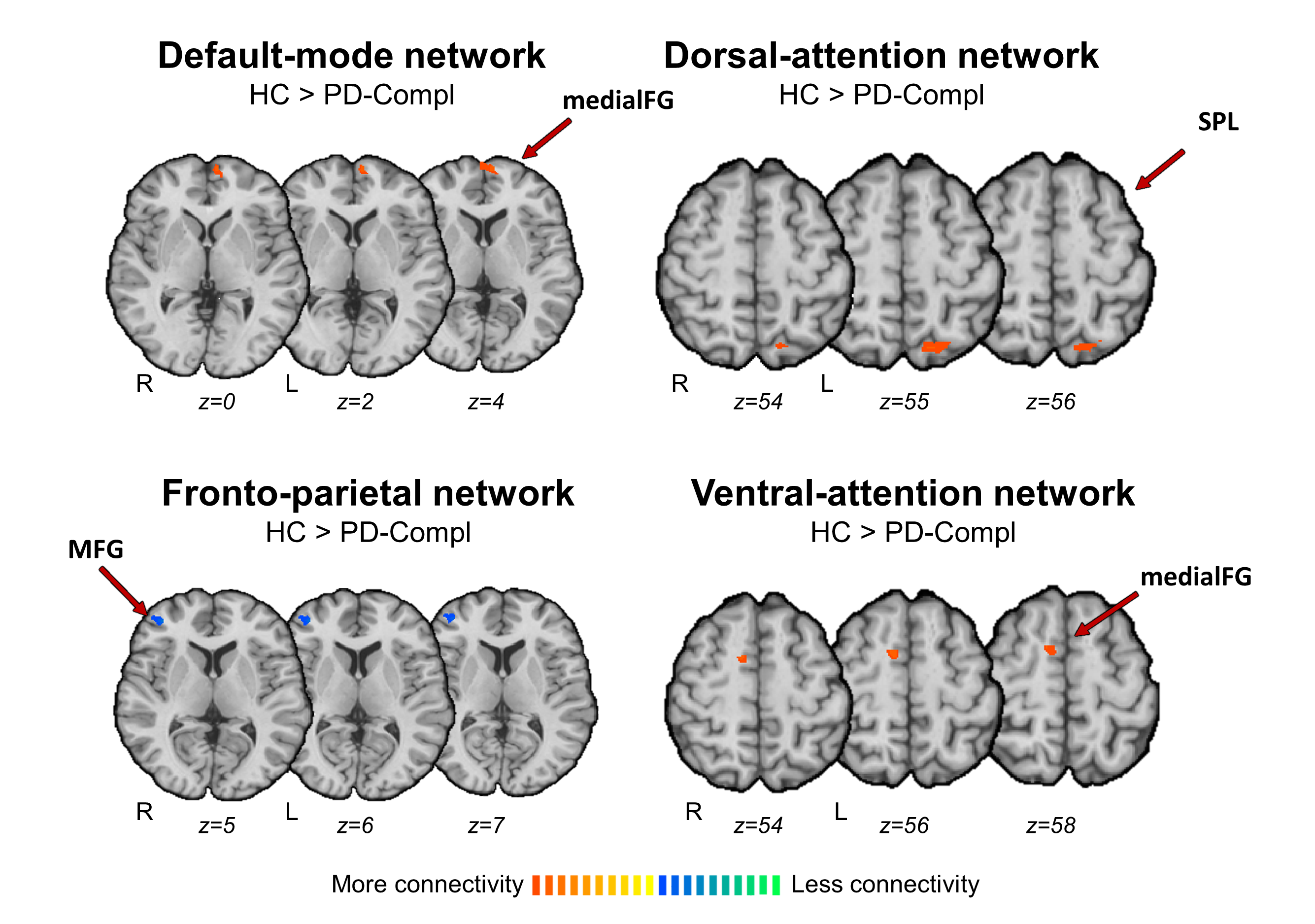

Supplement: Supplementary file 1 — Supplementary file1 Supplementary figure 1 Resting-state network connectivity changes in PD patients and controls. Whole-brain significant connectivity differences between PD-Compl and healthy controls. Cold colors represent less, and hot colors represent more connectivity. Abbreviations: R: right; L: left; MFG: middle frontal gyrus; medialFG: medial frontal gyrus; SPL: superior parietal lobule (TIF 2286 KB) [file 415_2023_12020_MOESM1_ESM.tif]

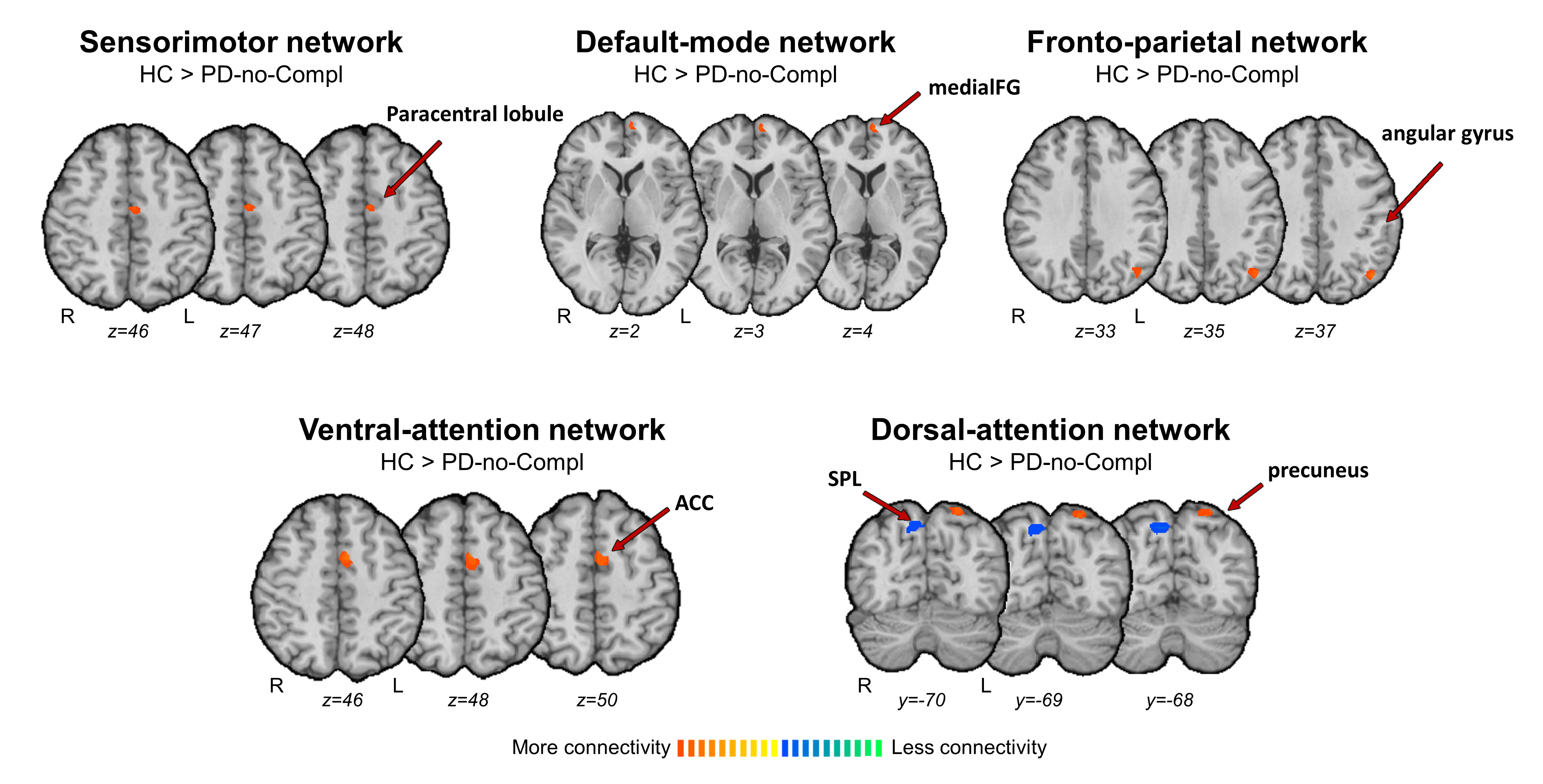

Supplement: Supplementary file 2 — Supplementary file2 Supplementary figure 2 Resting-state network connectivity changes in PD patients and controls. Whole-brain significant connectivity differences between PD-no-Compl and healthy controls. Cold colors represent less, and hot colors represent more connectivity. Abbreviations: R: right; L: left; medialFG: medial frontal gyrus; ACC: anterior cingulate cortex; SPL: superior parietal lobule (TIF 2958 KB) [file 415_2023_12020_MOESM2_ESM.tif]
